# Geriatric Oncology as an Unmet Workforce Training Need in the United Kingdom—A Narrative Review by the British Oncology Network for Undergraduate Societies (BONUS) and the International Society of Geriatric Oncology (SIOG) UK Country Group

**DOI:** 10.3390/cancers15194782

**Published:** 2023-09-28

**Authors:** Emma G. Khoury, Thitikorn Nuamek, Sophie Heritage, Taylor Fulton-Ward, Joanna Kucharczak, Cassandra Ng, Tania Kalsi, Fabio Gomes, Michael J. Lind, Nicolò M. L. Battisti, Kwok-Leung Cheung, Ruth Parks, Jessica Pearce, Mark A. Baxter

**Affiliations:** 1 Academic Cancer Sciences Unit, University of Southampton, Southampton SO16 6YD, UK; 2 Medical Oncology, The Christie NHS Foundation Trust, Manchester M20 4BX, UK; thitikorn.nuamek@nhs.net (T.N.);; 3 Cambridge University Hospitals, Cambridge CB2 0QQ, UK; 4 Institute of Immunology and Immunotherapy, College of Medical and Dental Sciences, University of Birmingham, Birmingham B15 2TT, UK; 5 School of Clinical Medicine, University of Cambridge, Cambridge CB2 OSP, UK; 6 Wythenshawe Hospital, Manchester University NHS Foundation Trust, Manchester M23 9LT, UK; 7 Department of Ageing of Health, Guy’s Hospital, Guy’s and St Thomas’ NHS Foundation Trust, London SE1 7EH, UK; 8 School of Life Course and Population Sciences, King’s College London, London WC2R 2LS, UK; 9 Queens Centre for Oncology and Haematology, Hull and East Yorkshire NHS Trust, Hull HU16 5JQ, UK; 10 Cancer Research Group, Hull York Medical School, University of Hull, Hull HU6 7RX, UK; 11 Breast Unit, Department of Medicine, The Royal Marsden NHS Foundation Trust, London SW3 6JJ, UK; 12 School of Medicine, University of Nottingham, Nottingham NG7 2UH, UK; 13 Leeds Institute of Medical Research at St James’, University of Leeds, Leeds LS2 9JT, UK; 14 Division of Molecular and Clinical Medicine, Ninewells Hospital and Medical School, University of Dundee, Dundee DD2 1SY, UK; 15 Tayside Cancer Centre, Ninewells Hospital and Medical School, NHS Tayside, Dundee DD2 1SG, UK

**Keywords:** geriatric oncology, medical education, undergraduate, postgraduate, workforce planning

## Abstract

**Simple Summary:**

People are more likely to develop cancer as they become older, and as people live longer, the number of older adults with cancer is steadily increasing. Managing cancer in older adults is challenging because they often have long-term conditions and wider needs that complicate treatment decisions and outcomes. However, the care of older adults with cancer is not formally taught during undergraduate medical education or postgraduate training in the United Kingdom. In this review, we provide an overview of the current education that medical students and training trainee doctors receive to prepare them for caring for older adults with cancer and highlight where challenges exist. We summarise the research conducted and strategies implemented internationally and use this knowledge to provide recommendations which may improve the education and training of doctors to meet the needs of older adults with cancer.

**Abstract:**

Cancer is a disease associated with ageing. Managing cancer in older adults may prove challenging owing to pre-existing frailty, comorbidity, and wider holistic needs, as well as the unclear benefits and harms of standard treatment options. With the ongoing advances in oncology and the increasing complexity of treating older adults with cancer, the geriatric oncology field must be a priority for healthcare systems in education, research, and clinical practice. However, geriatric oncology is currently not formally taught in undergraduate education or postgraduate training programmes in the United Kingdom (UK). In this commentary, we outline the landscape of geriatric oncology undergraduate education and postgraduate training for UK doctors. We highlight current challenges and opportunities and provide practical recommendations for better preparing the medical workforce to meet the needs of the growing population of older adults with cancer. This includes key outcomes to be considered for inclusion within undergraduate and postgraduate curricula.

## 1. The Ageing Population and Cancer

The general population is ageing, which has significant implications for its health and well-being and the provision of age-attuned health services [1]. Globally, the number of people aged over 60 years will double by 2050 [2]. In the United Kingdom (UK) and worldwide, the incidence of cancer is higher in the older population [3,4]. Currently, those aged 75 and older account for 36% of new cancer cases in the UK and this figure is expected to rise to 46% by 2034 [5].

As ageing represents a major unmodifiable risk factor for different diseases, the number of older adults with multiple co-morbidities and chronic illnesses, such as cancer, is rapidly increasing [6]. A decline in functional status or ability to perform everyday activities is also observed with the latter stages of the ageing process and/or ill health [7]. Functional decline and accumulation of co-morbidities correlate with poorer treatment tolerance and cancer outcomes [8,9]. Given the interplay of cancer and ageing and the complexities of treating cancer in older adults, the field of geriatric oncology is gaining increasing momentum.

Geriatric oncology is a field of medicine focused on the care of older adults with cancer. Almost all healthcare professionals have a role in caring for older adults with cancer at some point in their careers. This role may involve the diagnostic, treatment, or recovery stage of the disease trajectory. As most cancer patients are older adults, every oncologist is a geriatric oncologist. Therefore, the cancer workforce must be equipped with basic skills in geriatric oncology to meet the needs of the ageing population, both nationally and globally.

The International Society of Geriatric Oncology (SIOG) is an international and multidisciplinary professional organisation aiming to strengthen health professionals’ capacity to care for older adults with cancer. To achieve this, SIOG has laid out key priorities for tackling the challenge, which feature clear goals for medical education [10]. The proposed strategies include integrating geriatric oncology into training programmes, promoting optimal clinical practice and meaningful research, providing focused educational materials, and organising formal educational activities for medical students and trainee doctors [10].

In this narrative review, we discuss the current landscape of geriatric oncology education and training in the UK, including the extent to which geriatric oncology is addressed in both undergraduate and postgraduate curricula, highlighting current challenges and opportunities. Considering these issues, we provide practical recommendations for better preparing the medical workforce within the UK to meet the needs of the growing population of older people with cancer. These recommendations are targeted at the different UK bodies who have an interest in medical training at an individual, loco-regional, national, and international level.

This narrative piece has been produced with input from undergraduates (medical students or future doctors) and postgraduates (medical and surgical doctors in training) involved in the British Oncology Network for Undergraduate Societies (BONUS) [11], as well as members of the SIOG UK country group (a multidisciplinary group of all SIOG members in the UK). This included input from oncology (surgical, medical, haematology, and radiation) and geriatric specialties. The focus is primarily on those in medical training in the UK. The principles discussed should be considered for other healthcare professionals, including nurses and allied healthcare professionals, however, this is outwith the scope of this paper.

## 2. Geriatric Oncology on the Global Agenda

Geriatric oncology involves multidisciplinary input to address the wider needs of individual older adults with cancer. Internationally, substantial progress has occurred in the development of geriatric oncology in recent decades. The World Health Organisation (WHO) has refocused its activities on promoting and coordinating the global fight against non-communicable diseases, including cancer [12].

Likewise, in 2015, the United Nations (UN) recognised non-communicable diseases as a major threat to sustainable development and adopted 17 sustainable development goals (SDGs) for the 2030 agenda. SDG 3 focuses on ‘health and wellbeing’ and aims to reduce mortality from cancer, as well as improving several targets pertinent to cancer care in older adults [13].

Furthermore, the founding of SIOG in 2000 marked a crucial milestone for geriatric oncology. The SIOG now consists of more than 1700 members in over 80 countries and implements programmatic activities via four strategic domains: education, clinical practice, research, and collaboration and partnerships, with a list of 12 priorities which align with the UN’s third SDG and echo the current challenges in the field of geriatric oncology [10].

The American Society of Clinical Oncology (ASCO) has also promoted the field of geriatric oncology [14], including the opportunity for trainees to undertake fellowships in both oncology/haematology and geriatrics (including dual accreditation) and the award of dedicated geriatric oncology fellowships since 1997. The Cancer and Aging Research Group (CARG) [15] and the European Organisation for Research and Treatment of Cancer (EORTC) [16] Older Adult Council are pioneering research in the field of geriatric oncology.

Importantly, a key priority is the integration of geriatric oncology into training programmes for healthcare professionals. This priority highlights key components that should be included in this training, including changes in cancer and patient biology and function with ageing, the role of geriatric assessment, and personalised approaches to management which include patient-generated goals of care. Methods to support this education, such as mentorship, and the need to adapt according to geographical location and resource availability are noted.

## 3. Geriatric Oncology in the United Kingdom

There are policies and initiatives which aim to promote the provision of geriatric oncology in several UK national health and professional organisations, cancer charities, and research institutes.

### 3.1. The National Agenda

Marking its 70th anniversary, the National Health Service (NHS) developed the NHS Long Term Plan in 2019, which addressed a concern about population ageing and identified a plan to improve cancer care over the next ten years [17]. This included the aim to deliver personalised care to everyone diagnosed with cancer through a comprehensive needs assessment and integration of clinical nurse specialists and other support workers. It is hoped this will encourage a substantial increase in resources and notable initiatives across the country in the coming years. In addition, the National Cancer Strategy ‘Improving Outcomes’ document identifies a need to ensure age alone is not a barrier to receiving suitable treatment options and proposes that treatment should be tailored to the needs of older patients [18].

Specifically for cancer, The 10-Year Cancer Plan (2022) identified older adults as a group vulnerable to inequalities in cancer identification secondary to the COVID-19 pandemic [19]. However, the recent government ‘Major Conditions and Diseases Strategy’ (2023) identifies cancer and dementia as key areas but did not mention geriatric oncology specifically [20].

### 3.2. Medical, Charity, and Research Organisations

At an individual organisation level, the British Geriatrics Society (BGS), a multidisciplinary professional society that promotes healthcare for older adults, established a Special Interest Group in Geriatric Oncology in 2015 to promote education and identify research and training opportunities. That same year, the cancer charity Macmillan also established an Expert Reference Group (ERG), which focused on the use of geriatric assessment tools across the nation in addition to education promotion and led to workforce recommendations [21]. Since then, members of these groups have characterised the workforce characteristics and interventions needed to support older adults with cancer in the UK [22,23].

Moreover, the Joint Collegiate Council for Oncology (JCCO), the collaboration between the Royal College of Physicians of London (RCPL) and the Royal College of Radiologists (RCR) representing medical and clinical oncologists in the UK, has been working on practical guidance related to frailty assessment and management in oncology.

Improving the care of older adults is a growing national research focus. In 2019, the National Cancer Research Institute (NCRI) set one of its priorities to better coordinate care for adults with complex needs and hosted a workshop on improving outcomes for older adults with cancer, which highlighted specific potential areas for research [24]. Lastly, the National Institute for Health and Care Research (NIHR) Integrated Academic Training programmes, which support healthcare professionals to gain skills and undertake research alongside their clinical training, have priority research themes on ‘health needs of older people’ and ‘multimorbidity at any age’ and have created themed calls for projects focusing on ‘frailty’ and ‘multimorbidity in older adults’.

Together, these initiatives highlight the recognition of the need to optimise the care of our older adults with cancer in the UK.

### 3.3. Geriatric Oncology in the UK: Current Practice

Geriatric oncology services have been increasing across the UK; however, they are heterogeneous in format [25]. Providing a geriatric oncology service is highly resource-dependent, and thus provision varies across the country based on workforce resources and relative pressures within local healthcare systems. For example, in prostate cancer, national audit data suggest that an oncogeriatric service assessing the fitness of older patients considered for chemotherapy or radiotherapy is only available in approximately 14% of Trusts in England and Health Boards in Wales [26].

Cancer care is coordinated independently according to geographic regions, and it also differs according to nation. In some regions, geriatric expertise forms part of the supportive service, whereas it may be fully integrated within the oncology activity in other regions [25]. Some may focus on a specific tumour site, whilst some on patients of a certain age. For example, in Brighton, there is a specific geriatric breast surgery clinic where a geriatrician and a breast surgeon jointly assess patients with breast cancer, whilst a new service called the Senior Adult Oncology Programme (SAOP) launched at The Royal Marsden in 2021 targets those above 70 years of age being considered for systemic anticancer therapy [27].

Although international guidance exists recommending that frailty is assessed in older adults being considered for chemotherapy [28], this is not routine in the UK. However, the Rockwood Clinical Frailty Scale (CFS) [29] has been increasingly used to identify frail patients who may benefit from the input of specialist geriatric or oncogeriatric services in specific geographic regions [30,31]. The upcoming national guidance from the JCCO may further support the more widespread use of frailty assessments and development of pathways for specialist input within the UK.

### 3.4. Geriatric Oncology in the UK: Medical and Surgical Training

Within the UK, national undergraduate and postgraduate curricula underpin medical and surgical training. Similar to other countries, oncology education is primarily delivered in relation to individual tumour sites or as part of other specialities as opposed to a multidisciplinary approach including geriatric input. Table 1 summarises how geriatric oncology is covered in UK medical undergraduate education and postgraduate training level [32,33,34,35,36,37,38,39,40,41,42,43,44,45,46,47,48]; outcomes relating to geriatric oncology are not well represented.

For undergraduates, the curriculum is set by individual medical schools based on the requirements and learning objectives highlighted in the ‘Outcomes for Graduates’, a framework provided by the General Medical Council (GMC) first published in 2009 [49]. These outcomes non-specifically reference an understanding of geriatric oncology. For example, Outcome 1 details knowledge of population health outcomes but does not explicitly state an understanding of the ageing population or the increasing prevalence of cancer. Outcome 2 discusses the ability to undertake an effective consultation with a patient, which may also be inferred to include older patients, as well as being able to communicate with patients efficiently. Finally, Outcome 3, which focuses on the doctor as a professional, identifies the responsibilities of working with older people.

Relating to assessment, the GMC Medical Licensing Assessment (MLA) includes a section on ‘Medicine of Older Adult’, in which cancer is not included as a condition of relevance [50]. Additionally, geriatric oncology is not included under the ‘Cancer’ section.

In 2018, the Outcomes for Graduates were updated to include more detail on patients with multiple comorbidities, many of whom would likely be older adults. The updated outcomes stated that graduates should have knowledge of dealing with frailty and long-term physical conditions, as well as dealing with patients approaching the end of life. Whilst beneficial additions, the outcomes fail to link geriatrics and oncology and do not explicitly state an understanding of how to manage the older patient with cancer. Currently, within the UK undergraduate curriculum, there is no link between the two specialties and little guidance to the delivery of geriatric oncology training. As curricula drive education and training, this may mean that graduates have little exposure to geriatric oncology during their time at medical school.

At a postgraduate level, each training programme has its own curriculum created by the Royal Colleges and approved by the GMC. This encompasses the foundation programme, core training (internal medical training, acute care common stem, core surgical training), and higher speciality training (e.g., oncology, haematology, surgery, geriatrics).

The foundation programme curriculum [33] requires doctors to be familiar with the principles of dealing with frail and/or older adults. However, it fails to link frailty with cancer. During internal medical training, exposure to geriatric medicine is required; however, cancer is not listed under the geriatrics section, and likewise geriatrics is not listed under the oncology section [34,35].

After foundation and internal medical training, the UK postgraduate medical curricula are slightly more specific in terms of their geriatric oncology outcomes (as indicated in Table 1) [32,40,41,42]; however, they often fail to highlight educational needs for the specific specialty and lack granularity as to the required outcomes.

Recognised subspecialties within geriatric medicine higher training exist: for example, ortho-geriatrics, for which trainees have equitable opportunity nationally to gain exposure and training. However, at present, there are inadequate resources and opportunities for this to be possible for oncogeriatrics.

For postgraduate surgical training, the situation is similar to medicine across general surgery and all subspecialties [37,38,43,44,45,46,47,48]. There is no specific mention of geriatric oncology and only references to cancer and older adults in a site-specific manner.

Understanding of geriatric oncology is also important for a wider range of medical specialties, particularly palliative medicine and general practice, as well as for other healthcare professionals, including nurses and allied healthcare professions who also have a fundamental role in caring for older adults with cancer. However, examination of these additional curricula is beyond the scope of this paper.

Summarising the existing curricula, education relating to cancer and geriatrics exists but does not overlap within the current UK educational and training structure. The education and training relating to oncology tends to be tumour-site-specific and specialty-specific in surgery, rather than taking a whole systems or person approach. Therefore, there is little education or training on cancer for geriatrics trainees or on geriatrics for oncology or surgical trainees. The ultimate result is little linkage between geriatrics and oncology in clinical practice.

### 3.5. Undergraduate and Postgraduate Experiences and Perspectives

In order to improve knowledge and confidence in managing older adults with cancer, medical students and postgraduate trainees should have exposure to and specific training in geriatric oncology. However, currently this is not happening in the UK and has been reported as a postgraduate training issue [51].

Postgraduate trainee experiences have been evaluated in several surveys; many trainees feel that they are not achieving adequate exposure to geriatric oncology. In a national survey of oncology specialty trainees, 66.1% of the respondents reported never receiving training on the particular needs of older adults with cancer, and 19.4% of trainees reported only receiving this training once [51]. In addition, only 27.1% were confident in assessing risk to make treatment recommendations for older adults with cancer compared with 81.4% being confident in treating younger patients [51]. This percentage dropped even lower to 10.2% when talking about older patients with dementia [51].

Similarly, another study exploring the competence and confidence of trainees working in gynaecological oncology found that 67% of trainees believed they had inadequate training in the perioperative management of older patients with or without frailty [52]. Most of these trainees valued closer collaboration with geriatrics when managing older patients. This further highlights the need for improved exposure and training within geriatric oncology at the postgraduate level.

While studies at an undergraduate level are lacking, it stands to reason that the issues that exist for postgraduates will also apply to those at an earlier stage of their career.

### 3.6. Current UK Initiatives

To begin to address the above, some initiatives have already commenced in the UK, including the development of learning resources, meetings, and fellowship opportunities. Online resources are being developed, and there are several excellent UK-relevant existing book resources [53,54].

Since 2016, the BGS has organised an annual event named OncoGeriatrics in collaboration with other leading organisations, including the Macmillan ERG, the RCR, and The Christie School of Oncology.

Relevant to the future workforce, there are very few schemes that promote a career in geriatric oncology; however, examples do exist. One fellowship is available within the NHS Guy’s and St Thomas’ NHS Foundation Trust Geriatric-Oncology Liaison Development (GOLD) team. This is an outpatient based oncogeriatric liaison service that provides comprehensive geriatric assessments for older persons undergoing treatment for cancer. Another fellowship example is the Senior Adult Oncology Programme (SAOP) at The Royal Marsden.

## 4. Geriatric Oncology: Learning from International Success

Research conducted internationally also recognises gaps and further emphasises the importance of including specific geriatric oncology educational needs in both undergraduate and postgraduate medical curricula. However, there are centres of excellence and international work and successes that can provide valuable inspiration for how geriatric oncology teaching and training could be developed in the UK. In addition, Section 4.11 of the recently published American Society of Clinical Oncology (ASCO)/European Society of Medical Oncology (ESMO) recommendations for a global Medical Oncology curriculum provides a blueprint for future educational development [55].

### 4.1. The Gap in Geriatric Oncology Services and Training Worldwide

Several international reviews and studies have outlined the lack of training and progress in geriatric oncology education worldwide, resulting in low clinician confidence. A review conducted in 2017 examined the gap in geriatric oncology education across multiple regions including North America, Asia, Australasia, Europe, and Latin America [56]. One of the main challenges in Asia, Africa, and the Middle East is that geriatric medicine is not recognised as a distinct speciality, and its inclusion is lacking in medical curricula across several countries. Thus, its incorporation into oncology teaching has been limited.

In the US, there is a lack of geriatric oncology education, with fewer than 25% of community oncologists feeling confident in managing geriatric patients [57], and strikingly, fewer than half of haematology and oncology fellows reported having had a single geriatric oncology lecture during their training [58]. Within the same cohort, only a quarter had access to a geriatric oncology clinic, and only 12% of trainees had been taught how to perform a geriatric assessment efficiently. Other US-based commentaries have similarly noted this gap and the importance of increasing access to geriatric oncology education [59,60].

In recognition of this challenge, there have been several successful ASCO initiatives to support geriatric oncology training in the US. These include geriatric oncology fellowships awarded by the Hartford Foundation (the first in 1997), the possibility of dual training in geriatric and oncology/haematology, the development of geriatric assessment guidelines [61], and a freely available resources platform [14].

Similar to the US, in Canada, a group of researchers investigated geriatric oncology training in radiation oncology programmes [62]. They found that half of the participants lacked confidence in managing older patients with multiple comorbidities, polypharmacy, functional and cognitive impairment, and challenging social circumstances. Strikingly, 71% of trainees believed that the incorporation of geriatric oncology learning outcomes into radiation oncology training would improve patient care standards. This is in keeping with the study in the UK [63].

A similar experience is seen in Asia and Australia. A survey in Singapore revealed that 90% of oncologists would value the introduction of geriatric oncology training and 61% of oncologists have never sought the advice of a geriatrician for treatment decisions [64]. In response to this article, an Australian oncology trainee reported their concerns for the lack of geriatric oncology training in Australia and difficulties in attempting to find relevant experience [65].

Again, similar to the UK, this is driven by a lack of exposure in medical school and during training. In Japan, a survey was conducted across all medical and graduate schools, alongside cancer hospitals, to understand the existence of geriatrics and geriatric oncology training across the country [66]. This found that out of 48 medical schools, 23 included teaching on geriatrics, and one on geriatric oncology. Five out of 42 graduate schools included geriatrics teaching, and none in geriatric oncology, whilst across all 151 cancer hospitals surveyed, none had a geriatric oncology service. The authors commented on the great need to promote geriatric oncology education and training across the country.

These findings are supported by a global multi-centre review that highlighted reduced confidence in caring for the older population by clinicians and trainees and cited that only about one-third of training programmes had incorporated geriatric oncology training into their curricula [67]. To address this gap, they discussed the importance of dissemination and publication of such curricula to all healthcare professionals to facilitate implementation elsewhere.

Overall, these studies demonstrate that like the UK, education and training in geriatric oncology is also lacking internationally, with an impact on confidence in managing older adults with cancer.

### 4.2. Examples of Best Practice—Development of a Curriculum and Training Programmes

Despite clear deficits in exposure to and training in geriatric oncology, there are some excellent examples of best practice, with several training programmes worldwide that have already been developed and evaluated with positive outcomes. These are at a local, national, and international level. However, examples within undergraduate education are lacking.

At a local level, an example is the MD Anderson Cancer Centre, which developed a geriatric oncology curriculum for haematology and oncology fellows after identifying this as an educational need [68]. The team assessed their oncology curriculum and tried to integrate geriatric principles. Geriatric assessment, pharmacology, and psychosocial knowledge and skills were the three identified areas of educational need through an interprofessional team approach. Although this is presently in the pilot stage, it could pave the way for others hoping to implement a similar approach.

Additionally, a study conducted in the US revealed the significant benefits of providing specific geriatric assessment recommendations to oncologists, spanning eight ageing-related domains such as polypharmacy, functional, and nutritional status, as well as physical performance [69]. This intervention led to a marked reduction in high-grade toxicities amongst older patients with advanced lung cancer who are receiving treatment compared to those receiving usual care, where no recommendations were provided. This highlights the importance of adopting geriatric-assessment-guided management, including the provision of education and counselling materials and regular toxicity checks, as an integral component for enhancing patient care, treatment, and quality of life. This approach should be integrated when developing oncology training programmes.

At a national level in Singapore, a formal geriatric oncology program was developed at the National Cancer Centre Singapore (NCCS) for oncology nurses and community physicians. Annual geriatric oncology workshops are organised for all healthcare professionals and the program has also been incorporated into medical oncology fellowship training at NCCS [70]. Geriatric oncology training programmes have also been developed for nurses and showed success [71,72], demonstrating that short courses can be beneficial for the wider healthcare team.

Internationally, as face-to-face educational channels became less feasible during the COVID-19 pandemic, SIOG and other professional societies, such as ASCO and ESMO, have been producing several electronic modules to target a broader population [73,74].

In addition, SIOG hosts an in-person Advanced Course in Geriatric Oncology annually in Treviso, Italy. Similar courses have also been started in Australia and India. They are all open to global candidates. This course is a valuable educational opportunity for upskilling the medical and broader healthcare workforce [75,76]. The course has been an enormous success, and has been referenced as an excellent contributor to improving geriatric oncology training globally [10,67,77]. However, the development of educational opportunities at a loco-regional and national level is essential to maximise access.

## 5. Recommendations

International guidance recommends that all older adults with cancer are screened for potential frailty and have access to geriatric assessments [28]. These assessments can identify and allow subsequent management of reversible frailty-related issues to improve patient outcomes. It is vital that current and future doctors receive training in how to assess for and manage frailty and co-morbidity in the context of cancer, and vice-versa [28,78,79].

Within the UK, geriatric oncology is not well represented in the current medical or surgical curricula. Consequently, medical students and doctors feel under-trained and under-prepared to provide age-attuned care to frail and/or older adults with cancer. There is, therefore, an urgent need for change to equip future doctors, especially oncologists, surgeons, and geriatricians, with skills to confidently manage the complexities of older adults with cancer. Relevant competencies for training that could be considered include the awareness, knowledge, and skills listed in the 2016 ESMO/ASCO recommendations for a global curriculum in Medical Oncology [55].

The need for change requires recognition, not only by educational bodies, but also healthcare leaders in the UK. Suggested recommendations at an individual, local, national, and international level to improve geriatric oncology education and training are provided in Table 2. Some of these can be achieved in the short term, but others are likely long-term aspirations.

The key in the short term is to increase the awareness and availability of educational material and opportunities in the field. There are already various educational and clinical resources that can support trainees and clinicians in upskilling and providing optimal care for frail and older people with cancer. These include international guidance, books, and courses.

At a local level, the GMC Outcomes for Graduates recommends that a proportion of the individual medical school curriculum should be student-selectable, i.e., the student has a choice. This provides scope for medical schools to encourage student-selected components (SCC) and increase the availability of special study modules (SSM) focusing on geriatric oncology without a direct impact on the curriculum. To achieve this, awareness and support in senior staff is required to enable these opportunities to be offered and become established.

At a national level, student or trainees’ groups and societies, such as the British Oncology Network for Undergraduate Societies (BONUS) [11] and the National Oncology Trainees Collaborative for Healthcare Research (NOTCH) [80], should organise regular events and courses. As mentoring can increase interest in the field [81], and BONUS has developed a national oncology mentorship scheme in the past, organising a similar scheme dedicated to geriatric oncology or including mentors from geriatric oncology in the current scheme are encouraged.

Furthermore, leading professional organisations, cancer centres, and dedicated charities, such as the SIOG UK Country Group, the BGS, the RCR, the Macmillan ERG, The Royal Marsden School, and The Christie School of Oncology, should be the main contributors to creating digitised learning modules and look for ways to organise international exchange or scholarship programmes to other countries like the US and France, where progress has already been made. Annual events, like OncoGeriatrics, should continue on a regular basis, and collaboration between organisations will help promote knowledge sharing and disseminate learning activities.

On a similar theme, currently available fellowships in Geriatric Oncology should continue and expand, although the availability of funding is a limiting factor.

In the long term, there is a need for evidence-based research to map the existing curriculum against the needs of geriatric oncology, aiming for formal incorporation into undergraduate and specialty training. This can be facilitated through the Medical Schools Council (MSC), followed by the GMC revision of Outcome for Graduates.

While we await this research, we must first recognise the link between oncology and geriatrics. This requires equipping the oncology and surgical disciplines with the professional knowledge, skills, and behaviour required for geriatric assessment. We also must accomplish this for the geriatric discipline in relation to key oncological skills. Future intended learning outcomes (ILOs) and capabilities should be developed around these principles.

An aspiration at a postgraduate training level is the emergence of geriatric oncology as a new discipline (like ortho-geriatrics), meaning oncology (including surgery, radiation, and medical/haematology) working with geriatrics, as well as the implementation and dissemination of a curriculum for geriatric oncology.

More work is needed to improve undergraduate education and postgraduate training in geriatric oncology globally. Although each healthcare system is unique, international successes and existing resources can be drawn from and utilised in the UK and beyond to help better prepare the medical workforce to meet the needs of the aging population.

## 6. Conclusions and Future Directions

We have identified challenges and gaps in undergraduate and higher training in relation to geriatric oncology in the UK, for which specific solutions are required. We have suggested recommendations at an individual (e.g., active participation in events and projects), local/regional (e.g., provision of experience or fellowships), national (e.g., research opportunities), and international level (e.g., exchange programmes). Collaboration with the geriatric oncology expert community is essential to help ensure that oncology educators and supervisors are adequately skilled in designing, delivering, and assessing geriatric oncology content. Only then can we start to make progress in improving the care of older people with cancer.

This issue is not only restricted to the training programmes for doctors, but also for nurses and allied healthcare professionals who equally play a crucial role in our multidisciplinary environment.

## Figures and Tables

**Table 1:** Contents related to Geriatric Oncology in the UK Medical and Surgical Training Curricula. ENT, Ear, Nose, and Throat; IMT, Internal Medical Training; JRCPTB, Joint Royal Colleges of Physicians Training Board; RCR, Royal College of Radiologists; ST, specialty training.

Curriculum	Level	Contents Related to Geriatric Oncology (Including Their Gaps)
**Undergraduate**		
Outcomes for Graduates [49]	Undergraduate (Medical School)	Learning objectives for cancer medicine are listed, but not in conjunction with the learning objectives for medicine of older adults. No reference is made to the field of geriatric oncology or older adults with cancer.
**Postgraduate—foundation**		
Foundation Programme Curriculum [33]	Foundation Training (F1, F2)	All foundation doctors must be familiar with the principles of dealing with frail elderly patients. No reference is made to oncology.
**Postgraduate—core training**		
JRCPTB Curriculum for Internal Medicine Stage 1 Training [34]	Internal Medical Training (IMT1-3)	Mandatory training in geriatric medicine is required. Cancer is not listed as a condition or an issue under the geriatrics section.
JRCPTB Curriculum for General Internal Medicine Training (Internal Medicine Stage 2) [35]	Combined Specialty Training (ST3+)	
The Intercollegiate Surgical Curriculum Programme for Core Surgical Training [36] Neurosurgery (run-through training) [37] Cardiothoracic surgery (run-through training) [38] The Royal College of Obstetricians and Gynaecologists Core Curriculum (run-through training) [39]	Core Surgical Training (CT1–2)	General principles of cancer care and management related to specific surgical discipline (i.e., colorectal, urological) with multidisciplinary team discussions including oncology. No specific mention of geriatrics.
**Postgraduate—higher specialty training**		
JRCPTB Geriatric Medicine Higher Training Curriculum [40]	Specialty Training (ST3+)	Liaison with oncology is encouraged, but mandatory education or exposure to patients with cancer is not essential.
JRCPTB Medical Oncology Higher Training Curriculum [32]		References are made to the increased risk of cancer and the impact of comorbidities and social complexities in older adults. Specific educational needs related to geriatric oncology are not addressed.
RCR Clinical Oncology Specialty Training Curriculum [41]		
JRCPTB Haematology Higher Training Curriculum [42]		
The Intercollegiate Surgical Curriculum Programme for General Surgery [43] Urology [44] ENT [45] Orthopaedics [46] Plastics [47] Oral and Maxillofacial [48]	Higher Surgical Training (ST3+)	Understanding of the basic principles of surgical oncology including prevention, risk factors, management (both surgical and non-surgical), and ways of evaluating cancer treatments for a range of cancers seen by the specialist surgeon. No specific mention of geriatrics in any curriculum.

**Table 2:** Our recommendations. ASCO, American Society of Clinical Oncology; BGS, British Geriatric Society; BONUS, British Oncology Network for Undergraduate Societies; CARG, Cancer and Aging Research Group; ERG, Expert Reference Group; ESMO, European Society of Medical Oncology; GMC, General Medical Council; JRCPTB, Joint Royal Colleges of Physicians Training Board; MSC, Medical Schools Council; RCR, Royal College of Radiologists; SIOG, International Society of Geriatric Oncology; UKFPO, UK Foundation Programme.

Recommendations
Individual Medical students, trainees, clinicians: engage with existing services providing specialised oncogeriatrics care, seek opportunities to undertake teaching/training in frailty and geriatric oncology; consider joining oncogeriatric special interest groups, e.g., BGS oncogeriatrics special interest group, SIOG/Young SIOG, CARG; utilise published guidance materials developed by national and international bodies focussing specifically on the optimal care of frail and older people with cancer. Local/regional Medical schools: include outcomes relating to geriatric oncology and frailty within curricula; offer student-selected components and special student modules focusing on geriatric oncology alongside offering taster weeks within the specialty. Hospitals and deaneries; include topics relating to geriatric oncology within local/regional training programmes and signpost and encourage trainees to utilise existing geriatric oncology and frailty training. Centres with expertise in oncogeriatrics, e.g., GOLD service, The Royal Marsden SAOP, The Christie SAO service: consider offering visits to trainees and clinicians interested in developing practical skills in oncogeriatrics. National National bodies relating to oncology and geriatrics, e.g., BGS, BONUS, NOTCH, JRCPTB, RCR: national oncology mentorship scheme, regular webinars on geriatric oncology, and creation of digitised learning modules; offer funding to support uptake of geriatric oncology training opportunities. Training bodies, e.g., MSC, GMC, UKFPO, JRCPTB, RCR: map existing curriculum against needs of geriatric oncology Research and academic training funders, e.g., NIHR: fund research and personal awards to support the development of geriatric oncology researchers. International SIOG, ASCO, ESMO, and CARG: collaboration between organisations and offering of international exchange or scholarship programmes, provide grants to support those interested in pursuing geriatric oncology training opportunities, develop and promote guidance materials specifically focussing on the optimal management of cancer in older people; use the availability of online learning modules and targeted events as reference points for clinicians.

## Data Availability

Not applicable.
